# Endovascular treatment of wide-neck saccular renal artery aneurysm with waffle-cone technique

**DOI:** 10.1590/1677-5449.200116

**Published:** 2021-05-10

**Authors:** Paulo Eduardo Ocke Reis, Guilherme de Palma Abrão, Leonardo Roever

**Affiliations:** 1 Universidade Federal Fluminense – UFF, Niterói, RJ, Brasil.; 2 Universidade Federal de Uberlândia – UFU, Uberlândia, MG, Brasil.

**Keywords:** aneurysm, endovascular, endovascular procedure, therapeutic embolization, stent, aneurisma, endovascular, procedimento endovascular, stent

## Abstract

In the past, treatment of visceral artery aneurysms (VAAs) was exclusively surgical. These aneurysms were rarely diagnosed in elective or emergency cases. Development of imaging techniques and endovascular procedures has changed the history of the therapeutic options for this pathology. Endovascular management of VAAs has arisen to advances in endovascular techniques and has achieved high efficacy.

## Resumo

No passado, o tratamento de aneurismas da artéria visceral (VAAs) era exclusivamente cirúrgico e raramente diagnosticado em casos eletivos ou de emergência. O desenvolvimento de técnicas de imagem e procedimentos endovasculares mudou a história das opções terapêuticas dessa patologia. O manejo endovascular de VAAs surgiu devido ao avanço das técnicas endovasculares, o qual apresentou uma alta eficácia.

## INTRODUCTION

In the past, treatment of visceral artery aneurysms (VAAs) was exclusively surgical, using open approaches, until development of imaging techniques and endovascular devices expanded the treatment options.^1^^,^ 2 The key to success is to choose the best technique for each case individually. Almost all patients are asymptomatic and the principal indication for treating VAAs is aneurysm size; 2.0 or 2.5 cm when not ruptured.^1^^-^ 5 These aneurysms are still rare and if they rupture the mortality rate is high, so the objective of treatment is to prevent rupture by excluding the aneurysm sac while saving branch patency.^1^^-^ 6 Coil embolization has emerged as a management method for these patients, offering high efficacy and low invasiveness.^1^^-^ 6 Techniques for treatment of wide-necked aneurysms using Stent-Assisted Coil Embolization (SACE) have demonstrated effectiveness in complex wide-necked bifurcation cerebral aneurysms and can also be employed in extracranial aneurysms.^1^^-^ 10 The goal of treatment is to prevent the aneurysm from expanding by excluding it from the arterial circulation, saving branches and patency and ensuring freedom from rupture or reperfusion.^1^^-^ 6 Geometry has been shown to predict need for adjuvant techniques in endovascular treatment of aneurysms.^11^^,^ 12 Wide-neck aneurysms aspect and dome-to-neck ratios < 1.2 mm or neck length > 4.0 mm, almost always require adjuvant techniques in addition to coil embolization, such as stent-assisted techniques or balloon remodeling techniques.^1^^-^ 12

## PART I - CLINICAL SITUATION

**Figure 1:**
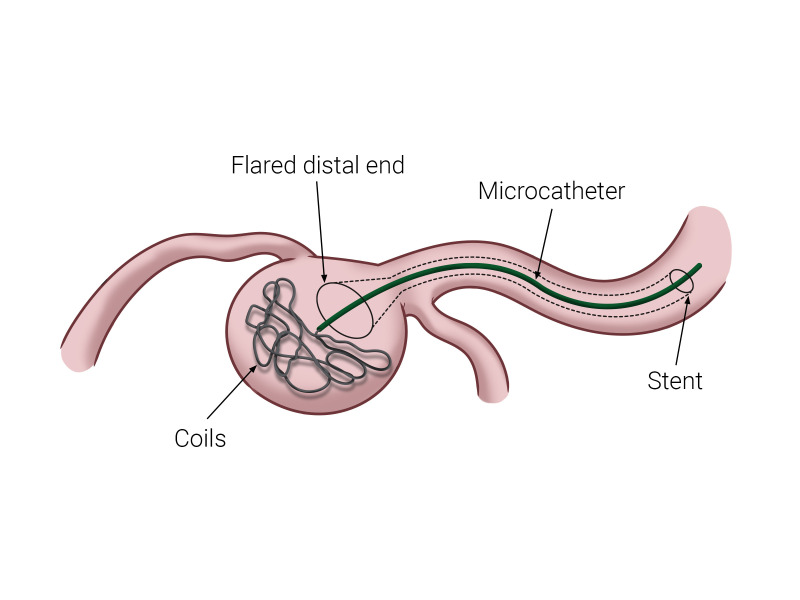
Schematic diagram illustrating a wide-necked saccular aneurysm treated with a Waffle-Cone Technique. Modified from Khattak et al.^11^

A 62-year-old woman presented with a saccular renal artery aneurysm. She was asymptomatic and diagnosis was the result of a finding during a regular clinical check-up. She had an intraparenchymal renal saccular aneurysm, measuring 2.7 x 2.0 cm and with a large aneurysm neck length (> 4mm), which would need a remodeling technique to exclude the aneurysm sac (Figure 1).

## PART II - WHAT WAS DONE

**Figure 2:**
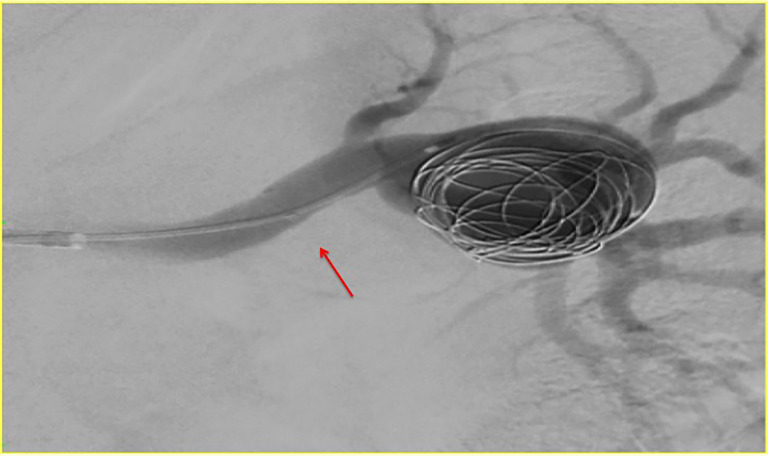
Microcatheter positioned inside the aneurysm sac, so we deployed the micro coils. Stent already deployed (red arrow).

**Figure 3:**
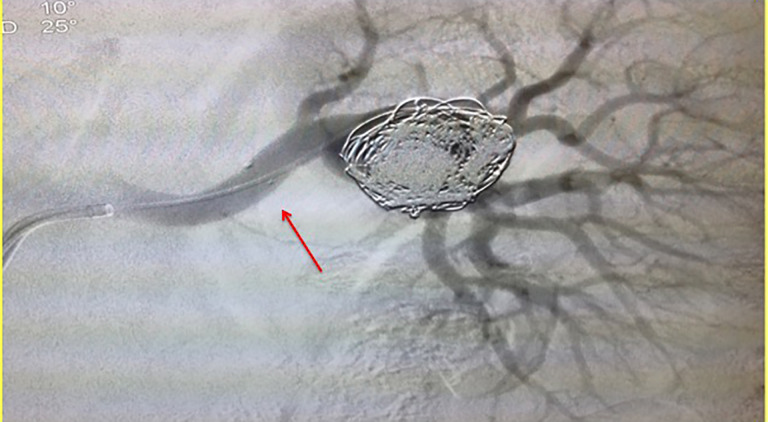
Final control arteriography. The red arrow indicates the radiopaque marking on the stent.

**Figure 4:**
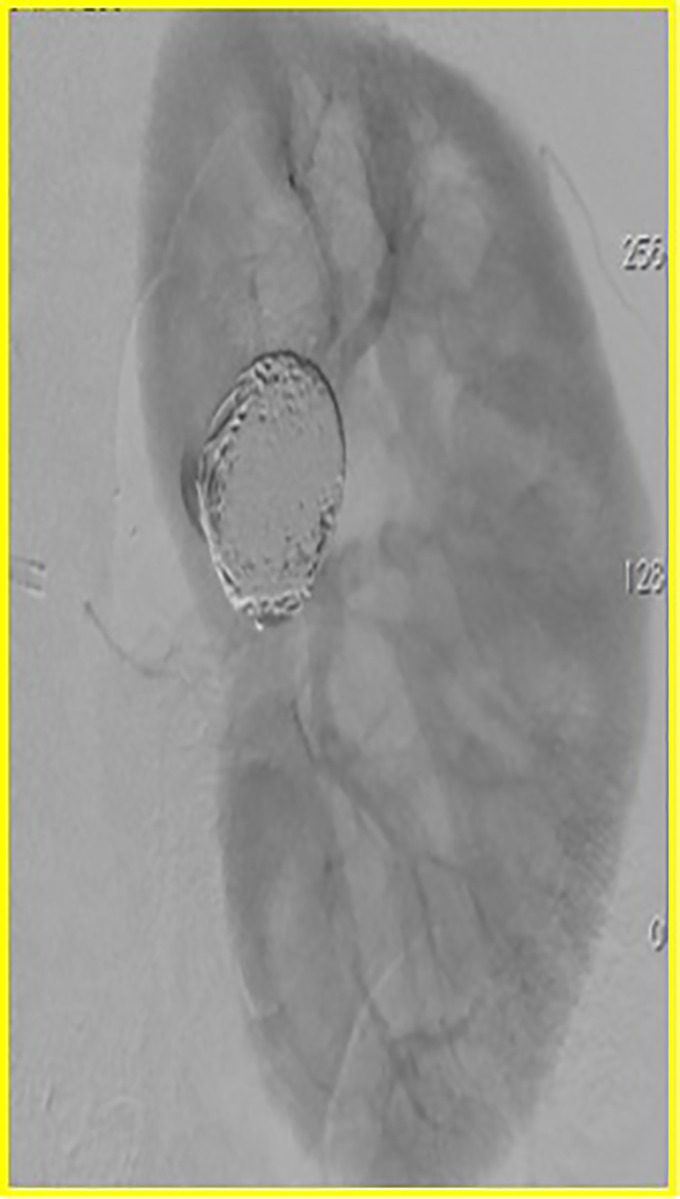
Late phase control arteriography showing complete exclusion of the aneurysm.

Our treatment approach was SACE using a waffle-cone technique^1^^-^ 10 (Figure 1). Access was obtained, the right common femoral artery was punctured using the Seldinger technique, and a 6Fr sheath was positioned in the left renal artery. A Prowler Select Plus^®^ microcatheter was then positioned inside the aneurysm and a 4.5 x 27 mm Enterprise^®^ (Cordis, Miami, FL, USA) self-expandable stent was implanted into the aneurysm, intentionally covering the bifurcation. Using a coaxial technique, a Prowler^®^ microcatheter was advanced into the aneurysm through the stent lumen for coiling (Figure 2). A total of 12 Trufill DCS Orbit coils^®^ were then deployed by controlled release. Digital subtraction angiography demonstrated complete occlusion of the aneurysm after stent assisted coiling (Figures 3 and 4).

**Figure 5:**
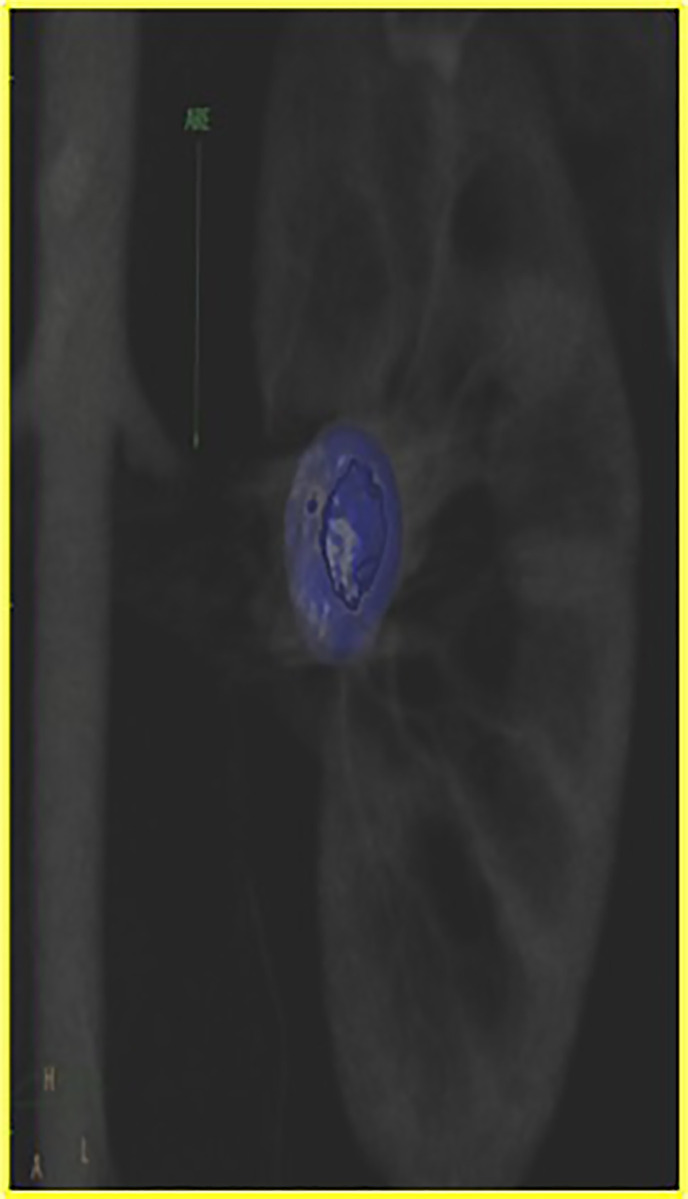
Coronal angiotomography with an algorithm to reduce metallic artifacts showing successful exclusion of an intraparenchymal renal saccular aneurysm. ARE = renal artery.

**Figure 6:**
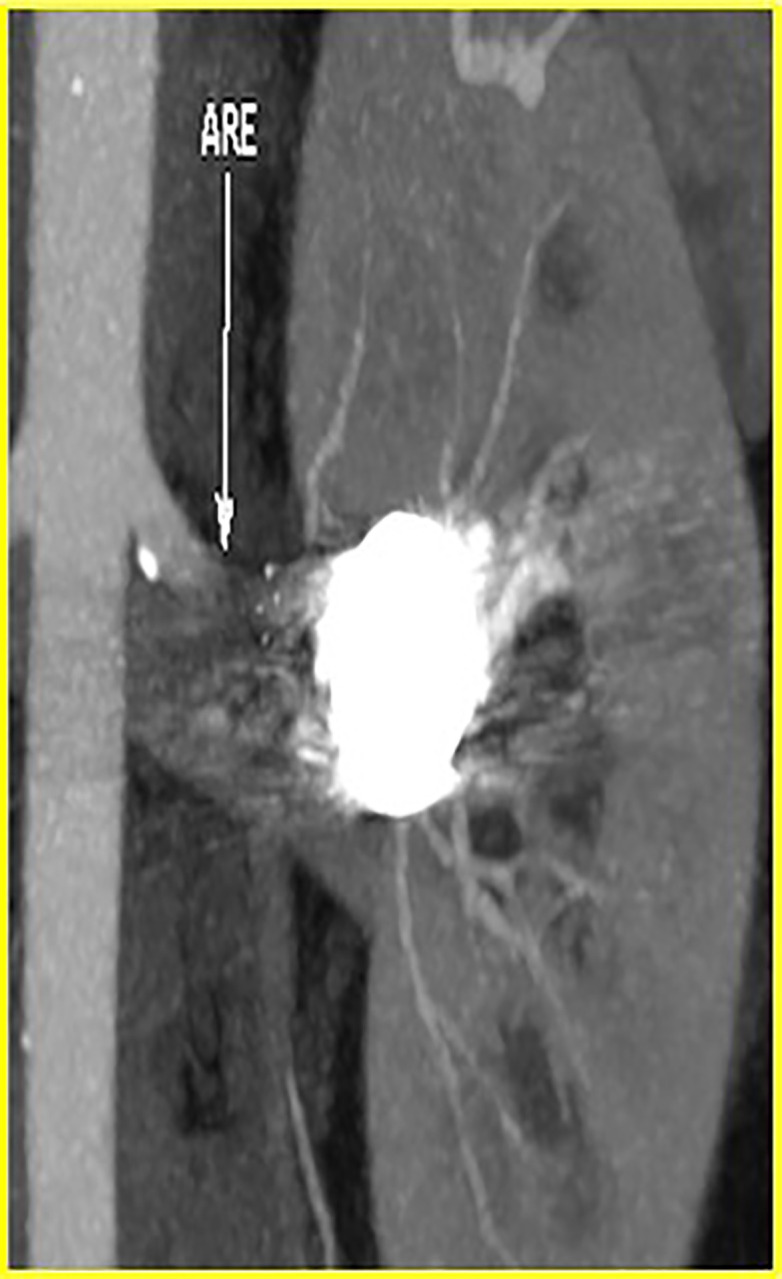
Coronal reconstruction of angiotomography with an algorithm to reduce metallic artifacts, showing complete exclusion of a 2.7 x 2.0 mm aneurysm sac using stent-assisted coil embolization. ARE = renal artery.

Final control arteriography demonstrated the aneurysm completely occluded (Figure 5). Control CT showed successful exclusion of a 2.7 x 2.0 cm saccular intraparenchymal renal aneurysm (Figure 6). The patient was given dual antiplatelet therapy after the procedure. She gave her consent to publication of her case details and images.

## DISCUSSION

Several stent-supported coiling techniques have been used to treat wide-necked bifurcation aneurysms, including waffle-cone constructs.^2^^-^ 5 First conceived in neuro-interventional practice, the differentiating factor with this technique is that it uses the stent as support to guide positioning of the coils to prevent prolapse during deployment and to help packing, resulting in a stable scaffold jailing the coils in the aneurysm sac.^2^^-^ 8 The diameter of the nitinol stent is determined based on assessment of the size of the aneurysm neck and the diameter of the proximal parent artery. The waffle-cone is thus created by deploying the stent’s proximal end within the renal artery and the distal end at the proximal extreme of the aneurysm. This placement avoids migration of the coils and allows good flow through the division branches. Liu showed that the waffle cone technique is an effective alternative tool for stent-assisted coiling of complex, wide-necked bifurcation aneurysms with unfavorable anatomies.^7^ Authors have determined criteria to define an approach to difficult aneurysms with aspect and dome-to-neck ratio <1.2mm, and a neck width >4.0 mm.^10^ In our decision, the ratio measurement determined during preoperative planning was the independent predictor for an adjuvant technique.

In conclusion, endovascular treatment of VAAs with stent-assisted coiling is a viable and safe procedure that is effective for treating complex wide-necked bifurcation renal artery aneurysms whose anatomic features are unfavorable for conventional coiling.^1^^-^ 5 The Waffle-Cone technique seems to be a technical option with effective arterial remodeling. However, long-term follow-up results are needed for evaluation.
